# 

**Published:** 1991-12

**Authors:** 


### West of England Medical Journal — Index 1991
### Volume 106

American Prisoners in Dartmoor ... ... ... 103

Angiotensin Activity, twenty years of investigating... 5

Anon ... ... ... ... ... ... 10

Appleton G ... ... ... ... ... ... 72

Atlanto-axial instability in Down's Syndrome ... 7

Bailey I. S. ... ... ... ... ... ... 61, 91

Banks R. A. ... ... ... ... ... ... 9

Barber S. G. ... ... ... ... ... ... 40

Bedding out ... ... ... ... ... ... 17

Bird J. ... ... ... ... ... ... ... 95

Book Reviews

Cardio-vascular System ... ... ... ... 81

Clinical Oncology ... ... ... ... ... 118

80 Years in Bath ... ... ... ... ... 118

Essentials of Uro-radiology ... ... ... 81

Essentials of Dermatology ... ... ... ... 45

Evolution and a creator ... ... ... ... 81

Lecture notes on the physics of radiology... ... 45

Second opinions in internal medicine ... ... 45

The best of Medical Humour ... ... ... 81

The return of blood to the heart ... ... ... 118

Bourns H. K. ... ... ... .. ... ... 11

Bristol Royal Infirmary as a Trust Hospital... ... 60

Burdens and Bristol ... ... ... ... ... 112

Burns-Cox C. ... ... ... ... ... ... 80

Burton J. L. ... ... ... ... ... ... 17, 77

Burton P.... ... ... ... ... ... ... 72

Byron, Lord, Pathos and Pathology ... ... ... 105

Celestin L. R. ... ... ... ... ... ... 105

Changing Face of Bristol Medicine, Symposium ... 30

Chest Physicians, why I like them ... ... ... 77

Childbirth, Lessons from the past ... ... ... 98

Clarke N. M. P. ... ... ... ... ... 33, 74

Crossley J. ... ... ... ... ... ... 96

Cobby M. ... ... ... ... ... ... 74

Cooke L. B. ... ... ... ... ... ... 7

Cooking the Books ... ... ... ... ... 79

Corner B. ... ... ... ... ... ... 94

Cornwall Helicopter, effect on ambulance response time 68

Davis J. D. ... ... ... ... ... ... 79

Discharge Summaries, towards better ... ... 40

Dover, Captain Thomas ... ... ... ... 96

Drowning, recreational, deaths from... ... ... 65

Dumoulin J. G. ... ... ... ... ... 10

Duncan A. ... ... ... ... ... ... 74

Dunn P. ... ... ... ... ... ... ... 13, 98

Early D. ... ... ... ... ... ... ... 110

Ehrlich, Paul and Almroth Wright ... ... ... 107

Epilepsy, Brain and Mind ... ... ... ... 95

Farndon J. R. ... ... ... ... ... .. 33

Fearn L. ... ... ... ... ... ... ... 5

Folley J. ... ... ... ... ... ... ... 9

Gillespie W. ... ... ... ... ... ... 107

Goble N. M. ... ... ... ... ... ... 15

Hamer A. J. ... ... ... ... ... ... 42

Hamilton W. ... ... ... ... ... ... 67

Hippocratic Oath, who wrote the? .. ... ... 91

How basic can you get? ... ... ... ... 115

Hunterian Lectures and Bristol — 1990 ... ... 33

James D. ... ... ... ... ... ... 43

Jancar J. ... ... ... ... ... ... ... 112

Job-sharing in General Practice... ... ... ... 67

Jones A. S. ... ... ... ... ... ... 72

Jones B. ... ... ... ... ... ... ... 101

King M. H. ... ... ... ... ... ... 40

Lansdall-Welfare R. ... ... ... ... ... 7

Leaper D.... ... ... ... ... ... ... 72

Leonardo Anatomical Drawings ... ... ... 99

Lousy story ... ... ... ... ... ... 10

Lunacy Bristol Fashion... ... ... ... ... 111

Lush B. ... ... ... ... ... ... ... 106

MacKenzie J. C. ... ... ... ... ... 5

Maintenance Dialysis in a District General Hospital 9

Menzies, Archibald, Surgeon Botanist ... ... 108

Michelman M. ... ... ... ... ... ... 44

Miller R. ... ... ... ... ... ... 33

N.H.S., Thoughts on ... ... ... ... ... 63

Naish J. ... ... ... ... ... ... ... 108

Nicholson S. ... ... ... ... ... ... 33

Nephro-calcinosis, hydration monitoring in... ... 15

Newborn in Antiquity, care of ... ... ... 94

Non-immune hydrops ... ... ... ... ... 43

Notes from Cornwall ... ... ... ... ... 115

Obituaries

Ralp Arthur John Baily ... ... ... ... 27

Herbert Kitchener Bourns ... ... ... ... 117

Kenneth Harvey Gaskell ... ... ... ... 27

Gerald Newton Golden ... ... ... ... 27

Osborne E. C. ... ... ... ... ... ... 5

Pancreas, neuro-endocrine tumour with salmonella cholangitis etc. ... ... ... ... 72

Parry, Caleb Hillier ... ... ... ... ... 101

Perforation of peptic ulcer in Hiatus Hernia into L Ventricle ... ... ... ... ... 42

Physicians for human rights ... ... ... ... 80

Private Practice — does it have a place? ... ... 61

Professional Society ... ... ... ... ... 79

Radial Head Dislocation, recurrent ... ... ... 44

Read M. D. ... ... ... ... ... ... 63

References for Medical Posts — how best to utilise them 11

Relations between Clinicians and Managers... ... 2

Roberts, Huw ... ... ... ... ... ... 110

Royal College of Surgeons visit to Bristol ... ... 16

Roylance J. ... ... ... ... ... ... 60

Rowse A.... ... ... ... ... ... ... 65, 68

Scott D. J. A. ... ... ... ... ... ... 33

Screening for Disease ... ... ... ... ... 37

Serendipity and Logic in Medical Research... ... 106

Sheffield E. ... ... ... ... ... ... 42

Sims P. ... ... ... ... ... ... ... 2

Skrabanek P. ... ... ... ... ... ... 37

Sluglett ... ... ... ... ... ... ... 92

Smoleniec J. ... ... ... ... ... ... 43

South Western Obstetrical and Gynaecological Society,

Meeting May 1991 ... ... ... ... ... 83

South West Orthopaedic Club,

Meeting in November 1990... ... ... ... 18

Meeting in April 1991 ... ... ... ... 51

Meeting in October 1991 ... ... ... ... 119

South West Radiologist's Association,

Meeting in October 1990 ... ... ... ... 20

Meeting in October 1990 ... ... ... ... 53

South West Urologist's Club, Meeting May 1990... 21

South West Vascular Surgeons,

Meeting in February 1990 ... ... ... ... 49

Meeting in February 1991 ... ... ... ... 85

Surgical Club of South West England,

Meeting in October 1990 ... ... ... ... 46

Trafford P. ... ... ... ... ... ... 103

Unwrapping of the Bristol Mummy ... ... ... 92

Ultrasound of the Infant Hip ... ... ... ... 74

Unauthorised version ... ... ... ... ... 10

Waldegrave Interview ... ... ... ... ... 58

Water Ram of Bristol and Neonatal Insufflation ... 13

Webb A. J. ... ... ... ... ... ... 16

Wessex Branch of the Association of Clinical

Pathologists, Meeting in December 1990... ... 23

Weston-super-Mare, History of Medical Services in 110

Which Journal? ... ... ... ... ... ... 80

Wilson M. G. ... ... ... ... ... ... 60, 99

**Editorial Committee**
Chairman of Editorial Board, Professor John Farndon
Editor—Mr. M. G. Wilson
Assistant Editors—Dr Paul Goddard, Dr Alison Round
Secretary—Mr Clive Ward
Member—Dr Kenneth Southgate

**Editorial Office**—*The Red House, 40 Coombe Lane, W.O.T., Bristol BS9 2BJ*
*Tel 0272 682320*

**Correspondents**
Dr Andrew Adam (Taunton)
Dr David Levine (Truro)
Dr Jack Davies (Bristol)
Professor Denis Pereira Gray (Exeter)
Dr Jose Jancar (Bristol)
Dr Michael Oliver (Barnstaple)
Mr Michael Reed (Gloucester)
Dr Michael Whitfield (Bristol)

**Associated Societies**
Bristol Medico-Chirurgical Society
Clinical Society of Bath
Severn Faculty of the Royal College of General Practitioners
South West of England and Wales Society of Dermatology
South West British Geriatric Society
South West Obstetricians' and Gynaecologists' Club
South West Orthopaedic Club
South West Paediatric Club
South West Radiologists' Association
South West Urologists' Club
South West Urological Oncology Group
South West Vascular Surgeons
Surgical Club of South West England
Tamar Faculty of the Royal College of General Practitioners
Wessex Association of Clinical Pathologists
West Country Chest Physicians' Club
West Country Physicians' Club

Members of the above organisations receive the journal as part of their membership subscription.
Annual Subscription Rate £25.00 including surface postage.

**Published by**
Clinical Press,
*Redland Green Farm,*
*Redland Green, Redland, Bristol BS6 7HF.*
*Tel 0272 237709/420256*

**Typesetting and Printing by**
*H. E. ILES (Central Press) LTD.,*
*49/51 Downend Road,*
*Kingswood, Bristol BS15 1SF.*
*Tel 0272 614161 (4 lines)*

**THE WEST OF ENGLAND MEDICAL JOURNAL acknowledges with gratitude the help it receives from NUFFIELD HOSPITALS**

### WEST OF ENGLAND MEDICAL JOURNAL

**DECEMBER 1991** **Volume 106(iv)**

